# Determinants and Dynamics of COVID-19 Vaccine Hesitancy in University Students: A Machine Learning Analysis

**DOI:** 10.3390/vaccines14050429

**Published:** 2026-05-11

**Authors:** Daliana Lobo Torres, Zahid Ahmad Butt

**Affiliations:** School of Public Health Sciences, University of Waterloo, Waterloo, ON N2L 3G1, Canada; dtorres@uwaterloo.ca

**Keywords:** COVID-19, booster vaccination, vaccine hesitancy, machine learning, SHAP, clustering

## Abstract

**Background:** Booster vaccine hesitancy poses a challenge to sustained COVID-19 immunization even among individuals who accepted primary vaccination. This study examined associated factors and patterns of change in vaccine attitudes among university students in Ontario, Canada. **Methods:** A cross-sectional survey dataset was analyzed using validated psychometric scales to measure hesitancy toward primary and booster COVID-19 vaccination. Changes in hesitancy were operationalized as the continuous difference between booster and primary scores (ΔVH). Gradient Boosting and XGBoost regression models were fitted to estimate ΔVH from demographic characteristics (age, gender, socioeconomic status), vaccination history, and attitudinal constructs including complacency, confidence in vaccine safety, and perceived necessity of vaccination. Predictor contributions were assessed using SHapley Additive exPlanations, and Gaussian Mixture Modeling was employed to identify latent profiles among students with increased hesitancy. **Results:** A substantial proportion of students demonstrated higher hesitancy toward booster doses. Attitudinal factors, particularly complacency and safety perceptions, were the most influential predictors of increased hesitancy, whereas sociodemographic characteristics showed limited influence. Three distinct profiles of booster hesitancy were identified, reflecting heterogeneous patterns of vaccine attitudes and behaviors. **Conclusions:** These findings suggest that booster hesitancy in the study population is primarily associated with modifiable perceptions and can be effectively characterized using machine learning approaches that may inform targeted public health communication strategies.

## 1. Introduction

Vaccination is one of the most effective strategies for preventing infectious diseases, reducing severe diseases, and decreasing mortality [1]. Despite widespread availability of the COVID-19 vaccine, hesitancy remains a global challenge that limits herd immunity and public health effectiveness [1,2]. Vaccine hesitancy, as defined by the World Health Organization, refers to the delay or refusal of vaccination despite access to vaccines and is recognized as one of the top ten global health threats [3]. Studies during the COVID-19 pandemic report hesitancy rates ranging from 30% to 85% worldwide, with booster doses showing substantial reluctance in several populations [4,5].

Vaccine hesitancy is a complex and multi-dimensional phenomenon shaped by psychological, behavioral, social, and contextual determinants [6,7,8]. Previous research has grouped these determinants into four domains: individual, disease-related, vaccine-specific, and environmental or contextual factors [9,10]. Individual-level characteristics, including age, sex, educational attainment, and income, are consistently associated with vaccination decisions, although the direction and magnitude of these associations vary by population and setting [11,12]. Other factors, such as trust in healthcare systems, perceived effectiveness of preventive measures, and a sense of social responsibility, also strongly influence vaccine intentions [9,13]. Disease-level determinants focus on perceived susceptibility and severity, with underestimation of risk or over-reliance on alternative protective strategies that contribute to hesitancy [14]. Vaccine-level factors, including concerns about safety, efficacy, and potential side effects, are particularly important for rapidly developed vaccines such as those for COVID-19 [15,16].

Hesitancy is often amplified for booster or multi-dose vaccine series due to cumulative concerns or increased sensitivity to adverse events [17,18]. Vaccine attitudes are dynamic and can evolve even among individuals who initially accepted primary vaccination. Previous experiences, perceived risks, and changes in social and informational environments can influence willingness to receive subsequent doses. In this study, we define *susceptibility* as the increase in hesitancy from primary to booster doses, providing a quantitative measure of how vaccination attitudes shift over time.

Despite considerable research on vaccine hesitancy, important gaps remain. Traditional statistical methods often struggle to detect complex, nonlinear relationships and interactions among multiple determinants simultaneously [19]. Moreover, many studies do not capture the temporal change of vaccine hesitancy, especially among individuals who have already received their primary vaccination series [20,21]. Although prior work, including our own, has examined determinants of vaccine hesitancy [10,22], our understanding of how hesitancy develops over time and which factors exert the strongest influence remains limited.

## 2. Materials and Methods

A cross-sectional survey of University of Waterloo (UW) students in Ontario, Canada, was conducted during the Winter 2024 term by [10]. All actively enrolled undergraduate (n = 34,398) and graduate (n = 6010) students were invited via institutional emails, departmental newsletters, and social media, with 4453 completing the survey.

Participants self-reported COVID-19 vaccination status and vaccine hesitancy using validated instruments [23,24,25,26,27,28,29]. Additional data included socio-demographics (age, gender, academic level, faculty, income, employment, marital status, immigration status), past vaccination behavior (e.g., influenza, HPV, meningococcal), psychological antecedents based on the 5C model (Complacency, Confidence, Calculation, Convenience, Collective Responsibility) [9], and contextual factors such as perceived mandates, communication influences, and COVID-19 experiences [10,30].

Vaccine hesitancy (VH) scores for both primary and booster doses were calculated following CVHS and PACV scoring protocols [23,24,25], with responses coded as 0 (non-hesitant), 1 (uncertain), or 2 (hesitant). Raw scores were summed (range 0–30) and linearly transformed to a 0–100 scale $\left({VH}_{100}\right)$, then dichotomized into non-hesitant (<50) and hesitant (≥50), allowing for analysis of hesitancy as both a continuous and categorical outcome [10].

The survey was designed with measures to prevent duplicate responses and to protect participant anonymity. It was initially tested with 33 students representing diverse genders, ages, and academic programs, using both in-person and online formats, to gather feedback on question clarity, ease of navigation, and comprehension; any issues identified were addressed through revisions. A subsequent pilot involved 600 undergraduate and graduate students recruited via email to evaluate recruitment procedures and survey administration, resulting in a 9.5% response rate (n = 57) and no significant procedural problems. The survey’s content validity was refined through multiple iterative reviews by the research team and the Statistical Consulting and Survey Research Unit at the University of Waterloo [10]. Ethical approval was granted by the UW Research Ethics Board (ORE 46023).

### 2.1. Definition of Susceptibility

Susceptibility to changes in vaccine hesitancy between the primary series and booster doses was operationalized as the continuous difference in vaccine hesitancy scores for each participant. Let $VH_{i}^{\left(primary\right)}$ and $VH_{i}^{\left(booster\right)}$ represent the vaccine hesitancy scores for participant *i* corresponding to the primary vaccination series and booster doses, respectively. Then, the change in vaccine hesitancy is defined as

$$\Delta VH_{i}=VH_{i}^{\left(booster\right)}-VH_{i}^{\left(primary\right)}$$

Positive values of $\Delta VH_{i}$ indicate an increase in hesitancy (the participant became more hesitant toward the booster), negative values indicate a decrease in hesitancy, and a value of zero indicates no change. This continuous measure captures the full spectrum of possible changes in vaccine hesitancy, allowing for analyses that reflect both increases and decreases in hesitancy across the sample. This continuous measure captures changes in vaccine hesitancy in either direction, supporting analyses of variability across the sample.

### 2.2. Participant Characteristics

A total of 4453 University of Waterloo students completed the survey and were included in this analysis. Changes in booster vaccine hesitancy were measured on a continuous scale (ΔVH), capturing both increases and decreases relative to the primary vaccination series. Across participants, the mean change in hesitancy was $\bar{\Delta VH}=12.18\pm 15.62$.

**Figure 1:**
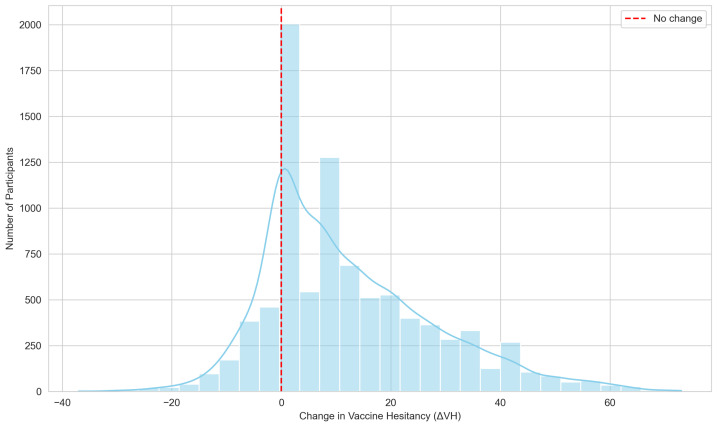
Distribution of changes in vaccine hesitancy (ΔVH).

Figure 1 presents this variation as a continuous distribution of ΔVH. The red dashed line at ΔVH=0 indicates no change in hesitancy, with values above this line representing increased hesitancy and values below indicating decreased hesitancy. The distribution exhibits a pronounced central peak around the interval 0–3, which contains the highest frequency of observations (n = 2004), reflecting that most participants experienced minimal changes in hesitancy relative to the primary vaccination series. A clear positive skew is observed indicating that a subset of participants reported increases in hesitancy. On the negative side, while the counts are lower, there are notable observations extending toward negative values, suggesting that some participants experienced meaningful decreases in hesitancy.

Table 1 complements Figure 1 by providing a more detailed, subgroup-level view of these changes for each demographic, academic, and socio-economic category.

Overall, the sample was predominantly young adults, with the majority aged 14–22 years (79.6% of non-susceptible and 85.9% of susceptible participants). Women were slightly more prevalent than men in both groups. Most participants were undergraduates, with fairly balanced distribution across years. Faculty representation was diverse, with Engineering, Health, and Mathematics accounting for the largest proportions. The majority of participants were domestic (and Canadian citizens or permanent residents) non-Indigenous students. Income distribution was heterogeneous, and marital status was predominantly single. Employment status was roughly balanced between employed and unemployed participants.

Preliminary comparisons suggest that certain demographic characteristics are associated with increase ΔVH. Younger participants (10–22 years) were slightly more prevalent in this group, and students in Engineering and Health faculties represented a slightly higher share. Gender, domestic status, and Indigenous identity were balanced across the two groups.

For subsequent analyses, a set of variables was included to characterize participants’ vaccine-related attitudes, perceptions, and behaviors across both the primary and booster vaccination phases. Attitudes toward vaccination were measured using individual survey items (Attitude_1_B, Attitude_2_B, Attitude_5_B for the booster phase; Attitude_1_P, Attitude_2_P for the primary phase), capturing participants’ beliefs and intentions regarding COVID-19 vaccination. Complacency-related constructs (Comp_At_B, Comp_At_P) reflect perceived necessity of vaccination, with higher values indicating lower perceived need for vaccination. These variables capture motivational aspects distinct from general vaccine attitudes. Safety perceptions (Safe_Score_B, Safe_Score_P) represent participants’ concerns regarding vaccine safety and potential side effects. Vaccination-related behavior was operationalized using Beh_Score_B, which reflects adherence to recommended vaccination practices. These variables were selected based on prior evidence indicating that vaccine attitudes, safety perceptions, complacency, and behavioral factors are key correlates of vaccine hesitancy [9,10,22]. Only independently defined scale variables were retained in the modeling framework.

To ensure the comparability of vaccine hesitancy scores across measurement occasions, a multi-group confirmatory factor analysis (CFA) was conducted to assess measurement invariance between the primary and booster contexts. The analysis evaluated configural and metric invariance. The configural model indicated that the same factor structure was identifiable across both contexts, with CFI = 0.843, TLI = 0.705, and RMSEA = 0.237. This establishes that the hypothesised latent structure is consistent across measurement occasions.

Metric invariance was then tested by constraining factor loadings to be equal across contexts. The constrained model showed no deterioration in model fit and instead exhibited slight improvements in comparative fit indices (CFI = 0.841, TLI = 0.762, RMSEA = 0.213), as well as improved information criteria (ΔAIC = −4.01; ΔBIC = −18.20). These results indicate that the relationships between indicators and the latent construct are equivalent across primary and booster measurements. Accordingly, the measurement instrument operates on a common latent scale across contexts, supporting the validity of interpreting ΔVH as variation in the underlying construct rather than as a measurement artefact.

### 2.3. Machine Learning Analysis

Changes in vaccine hesitancy between the primary vaccination series and the booster dose were quantified for each participant using a continuous measure. This approach preserves the full variability in individual responses and captures gradual shifts in attitudes that would be obscured by dichotomous classifications (e.g., hesitant vs. non-hesitant). The outcome (ΔVH) is defined as a within-individual change in vaccine hesitancy rather than a static construct. Accordingly, the model is designed to capture heterogeneity in changes in hesitancy across individuals, rather than to recover or reconstruct the underlying survey constructs.

Supervised machine learning regression models were applied, including Gradient Boosting Regressor (GB) and Extreme Gradient Boosting (XGBoost). These models characterize both linear and nonlinear relationships between predictors—such as demographic characteristics, prior vaccination behavior, psychological factors, and perceptions of COVID-19 risk—and changes in hesitancy. The performance of the model was assessed using metrics appropriate for continuous outcomes, including the mean squared error (MSE), mean absolute error (MAE) and the proportion of explained variance ($R^{2}$). The Gradient Boosting model was configured with 200 decision trees, a learning rate of 0.05, and a maximum tree depth of 3 to control model complexity and reduce overfitting. The XGBoost model used 200 boosting rounds, a learning rate of 0.05, and a subsample ratio of 1.0, meaning each tree was trained on the full dataset. Both models were optimized using the mean squared error (MSE) objective.

SHapley Additive exPlanations (SHAP) values were used to examine the contribution of individual predictors. This approach provides an estimate of how each variable influences changes in hesitancy at the individual level and identifies factors associated with higher or lower ΔVH values. SHAP values were computed using TreeExplainer algorithm to provide a global post hoc explanation of feature importance based on average marginal contributions of each feature across all samples.

Unsupervised clustering was also performed to identify student typologies exhibiting different patterns of change in vaccine hesitancy. This approach reveals naturally occurring subgroups emerged from shared combinations of highly influential predictors identified through the SHAP analysis. Clustering complements predictive modeling by highlighting latent population structures that support the identification of high-risk profiles.

## 3. Results

### Predictive Modeling of Booster Susceptibility

GB and XGBoost were applied to relate demographic, behavioral, vaccine-related attitudes, perceived safety, complacency, and behavioral scores to observed changes in hesitancy. We applied 5-fold cross-validation to evaluate model performance. We partitioned the dataset into five subsets, iteratively training on the fourth and testing on the fifth.

Cross-validation results (Table 2) indicate strong performance across all models. Both models produced high predictive accuracy across 5-fold cross-validation. These results indicate that most variation in changes in hesitancy can be captured by the selected predictors.

**Figure 2:**
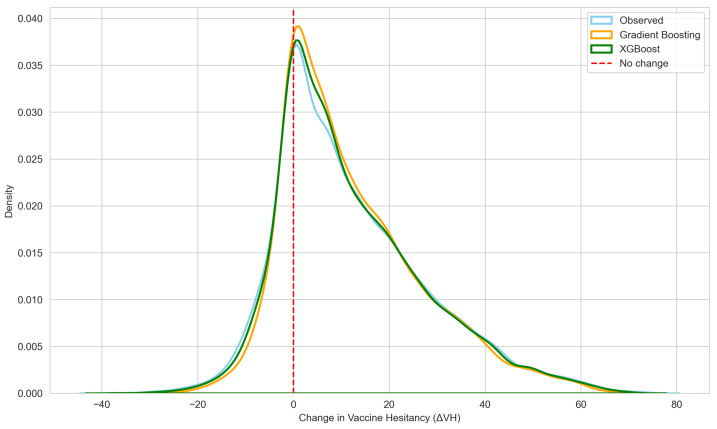
Visual comparison between the observed distribution of changes in vaccine hesitancy (ΔVH) and the distributions predicted by the Gradient Boosting and XGBoost models.

Figure 2 presents a visual comparison between the observed distribution of chan-ges in vaccine hesitancy (ΔVH) and the distributions predicted by the GB and XGBoost models. The curves represent kernel density estimates (KDEs), which provide a smoothed view of the distribution, highlighting where most students’ ΔVH values are concentrated and how widely the changes are spread. The KDEs suggest that both models generally capture the overall shape of the observed distribution, although small discrepancies remain, particularly near the zero-change region.

Agreement between observed and predicted changes in vaccine hesitancy (ΔVH) was evaluated using distributional statistics, correlation coefficients, and percentile-based comparisons for both GB and XGBoost models. Both models exhibited minimal systematic bias, with near-zero mean and median differences between predicted and observed values (GB: mean = −0.002, median = −0.478; XGBoost: mean = 0.006, median = −0.419) and negligible effect sizes (Cohen’s d≈0). Strong concordance was observed in rank ordering, indicating accurate reproduction of individual-level variation in hesitancy change. The GB model achieved Pearson and Spearman correlations of r=0.980 and $r_{s}=0.970$, respectively, whereas XGBoost achieved slightly higher concordance (r=0.989, $r_{s}=0.982$). Distributional alignment was further supported by percentile-based comparisons. Both models closely approximated the lower, central, and upper regions of the observed distribution (10th percentile: observed = −3.00, GB = −2.19, XGBoost = −3.07; median: observed = 9.00, GB = 8.52, XGBoost = 8.58; 90th percentile: observed = 34.00, GB = 33.41, XGBoost = 34.15), with XGBoost demonstrating marginally greater accuracy at the distribution tails. These results are consistent with the quantitative performance metrics reported in Table 2 and the distributional patterns illustrated in Figure 2.

**Figure 3:**
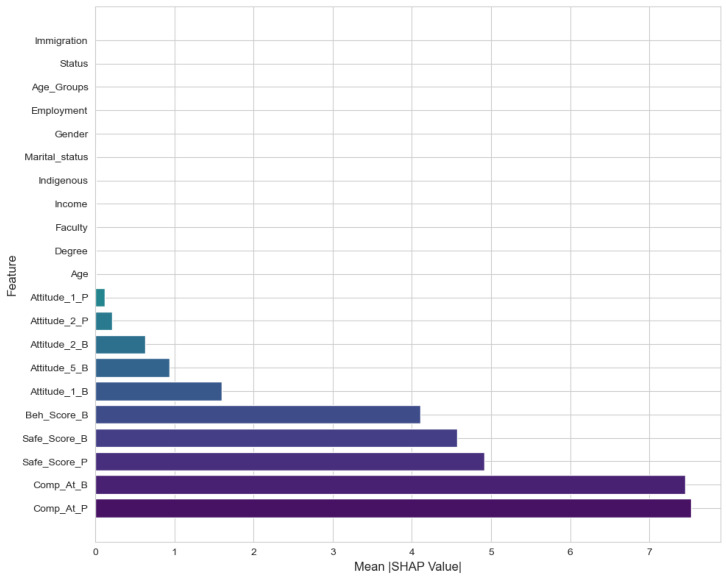
Overall SHAP values for predicting booster susceptibility.

SHAP values provided insight into how individual variables contributed to changes in ΔVH, highlighting both factors associated with increases in hesitancy and factors associated with decreases. SHAP values were calculated from the best-performing XGBoost model to quantify the contribution of each predictor to booster susceptibility. Figure 3 presents the mean absolute SHAP values for all variables, with higher values reflecting greater influence on the model’s predictions.

As shown in Figure 3, the model is primarily driven by vaccine-related psychological and behavioral constructs, with complacency, safety perceptions, and behavioral adherence emerging as the dominant contributors to predicted changes in booster hesitancy. Complacency variables (Comp_At_P and Comp_At_B) exhibit the highest mean absolute SHAP values, indicating that perceived necessity of vaccination constitutes the most influential dimension in explaining variability in hesitancy change. This is followed by safety perception measures (Safe_Score_P and Safe_Score_B), which capture concerns regarding vaccine safety and potential adverse effects, underscoring their consistent role in shaping booster-related decision dynamics. Behavioral factors (Beh_Score_B) also contribute to the predictive structure, albeit with comparatively lower magnitude, suggesting that reported vaccination-related behaviors provide additional but secondary explanatory information beyond attitudinal constructs. Demographic variables—including age, faculty, degree, income, marital status, employment status, gender, immigration status, and Indigenous identity—exhibit near-zero SHAP values (all < 0.01), suggesting minimal contribution to the predictive model relative to psychological and behavioral factors.

## 4. Student Profiles with Increased Vaccine Hesitancy

To examine patterns of increased vaccine hesitancy following the primary COVID-19 vaccination series, Gaussian Mixture Modeling (GMM) was applied to students with positive changes in hesitancy (ΔVH>0), using variables identified as the strongest predictors in the SHAP analysis. The Bayesian Information Criterion (BIC) indicated that three profiles best captured the heterogeneity among students.

**Figure 4:**
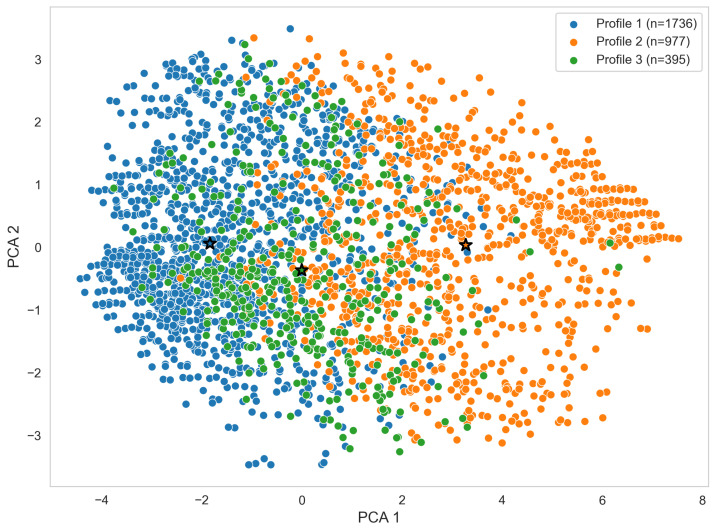
PCA scatter plot of students with increased vaccine hesitancy, colored by GMM-derived profiles. Cluster centroids are indicated by stars.

Figure 4 displays the distribution of participants with increased vaccine hesitancy along two principal components. Cluster centroids are indicated by stars, illustrating the separation between subgroups in two-dimensional space. Legend annotations report the number of participants in each profile, highlighting differences in cluster size and the underlying heterogeneity within this population.

**Figure 5:**
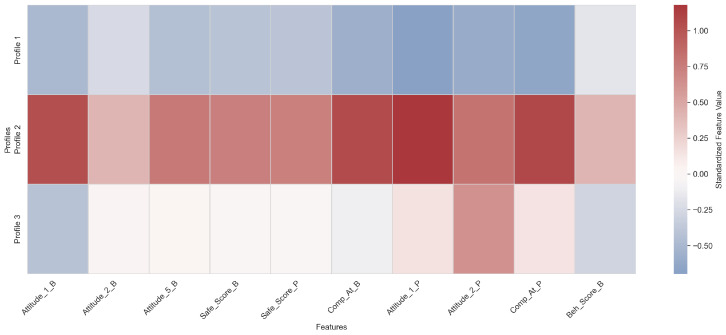
Heatmap of standardized feature means for GMM-derived profiles of students with increased vaccine hesitancy. Higher values indicate stronger hesitant responses in attitudes, safety perceptions, complacency, and vaccination-related behaviors.

Figure 5 presents a heatmap of standardized feature means for each profile. Higher values correspond to stronger hesitant responses, while lower values indicate lower hesitancy. All measures were standardized to allow for comparison across variables with different scales. This figure allows public health practitioners to quickly identify which attitudes, safety perceptions, or behaviors distinguish each profile.

Profile 1 is characterized by consistently negative standardized scores across all measured constructs, including attitudes (Attitude_1_B, Attitude_2_B, Attitude_5_B, Attitude_1_P, Attitude_2_P), safety perceptions (Safe_Score_B, Safe_Score_P), complacency (Comp_At_B, Comp_At_P), and vaccination behavior (Beh_Score_B). This pattern reflects a coherent behavioral phenotype characterized by generally low levels of vaccine hesitancy-related constructs across both vaccination phases, with limited deviation between primary and booster-related measures. This profile represents the largest subgroup of students, as illustrated in Figure 4.

Profile 2 exhibits uniformly positive standardized scores across all constructs, indicating elevated levels of hesitant attitudes, increased complacency, greater safety concerns, and higher behavioral resistance to vaccination across both primary and booster phases. This configuration reflects a consistent high-hesitancy behavioral phenotype, characterized by strong alignment across cognitive, perceptual, and behavioral dimensions of vaccine hesitancy. This profile constitutes the second largest subgroup in the sample.

Profile 3 demonstrates a heterogeneous or mixed configuration, with near-neutral to slightly positive values in selected primary-phase attitudinal measures (e.g., Attitude_2_P, Comp_At_P) and lower or slightly negative scores in several booster-phase constructs (e.g., Attitude_1_B, Safe_Score_B, Beh_Score_B). This pattern suggests partial dissociation between primary and booster-related responses, reflecting an intermediate behavioral phenotype characterized by inconsistent or context-dependent shifts in vaccine hesitancy across phases. This profile represents the smallest subgroup in the sample.

This clustering analysis provides a segmentation of the population into distinct profiles that may inform targeted public health interventions, rather than treating vaccine hesitancy as a uniform phenomenon across individuals. Profile 2, with high scores across all measures, identifies the students who are at highest risk of persistent hesitancy and may benefit most from targeted public health interventions, such as tailored communication and educational campaigns. Profiles 1 and 3, with lower or mixed scores, represent students with minor or intermediate increases, suggesting varying levels of engagement and potential receptiveness to interventions.

The identified profiles represent patterns within the ΔVH>0 subgroup. Additional analyses including the remaining population were performed and are presented in Appendix A.

## 5. Discussion

This study examined changes in COVID-19 booster vaccine hesitancy among university students by applying machine learning regression and clustering to identify the key predictors of change and to characterize heterogeneous response patterns, respectively. Unlike traditional cross-sectional analyses, we operationalized vaccine hesitancy change (ΔVH) as the continuous difference between hesitancy toward primary vaccination and booster doses, thereby capturing shifts in attitudes over time.

A key contribution of this study is the integration of a change-based analytical framework with interpretable machine learning to model within-individual dynamics in vaccine hesitancy. In contrast to prior work focused on static associations, our approach captures temporal variation between primary and booster vaccination, enabling the examination of how psychological constructs evolve over time. Moreover, the combination of nonlinear predictive modeling, SHAP-based interpretability, and unsupervised clustering provides a unified framework that not only predicts changes in hesitancy but also identifies heterogeneous behavioral pathways underlying these changes. Together, this multi-level perspective advances a dynamic and structured understanding of vaccine hesitancy beyond static risk-factor models.

Our findings highlight the underlying psychological structure of vaccine hesitancy change, suggesting that shifts in attitudes reflect dynamic cognitive re-evaluation processes occurring between vaccination phases. From a theoretical perspective, these findings are consistent with the Health Belief Model and broader risk perception frameworks, which posit that vaccination decisions are shaped by perceived susceptibility, perceived severity, and perceived barriers [14,31]. In this context, complacency can be interpreted as a reduction in perceived disease threat or vaccination necessity, while safety concerns reflect perceived barriers related to potential adverse effects. The observed changes in hesitancy between primary vaccination and booster uptake likely reflect temporal shifts in these cognitive evaluations as pandemic urgency decreases and individuals are exposed to evolving information environments.

The distribution of ΔVH was positively skewed, indicating that a substantial subset of students exhibited increased hesitancy after the primary series. This observation aligns with prior studies documenting that booster doses frequently elicit greater reluctance compared with initial vaccine uptake [4,5,17].The pronounced central peak near zero indicates that the majority of students experienced little to no change in their vaccine attitudes. However, the spread of values around this peak reveals different variation in both the direction and magnitude of change, highlighting the importance of tailoring public health strategies to address these heterogeneous patterns of booster hesitancy.

Machine learning models, particularly XGBoost, provided strong predictive performance ($R^{2}$ = 0.977), demonstrating that changes in vaccine hesitancy can be captured through nonlinear interactions among psychological and behavioral variables rather than simple additive effects. SHapley Additive exPlanations (SHAP) indicated that complacency during both primary and booster phases was the most influential predictor, followed by composite attitude scores and safety perceptions. These results align with the established literature on vaccine confidence, which consistently identifies perceived necessity and risk evaluation as central determinants of vaccine acceptance [9,10,13]. Importantly, the dominance of these modifiable constructs underscores their relevance for intervention design, particularly in shifting behavioral outcomes during booster campaigns.

While SHAP analysis identifies the relative importance of individual predictors, the clustering approach captures how these predictors co-occur within individuals, revealing distinct multivariate patterns of attitudes, safety perceptions, and behaviors that are not observable through feature importance rankings alone. Profile 2, with uniformly high standardized scores for attitudes, safety concerns, and complacency, comprised a group at heightened risk for persistent hesitancy. Profile 1 exhibited minor changes in hesitancy and relatively low baseline negative attitudes, indicating stability in vaccine acceptance. Profile 3 presented mixed patterns, suggesting situational hesitancy that may respond to context-specific messaging or support. These heterogeneous patterns echo findings from other vaccine hesitancy research showing that hesitancy is not monolithic but instead reflects a spectrum of behavioral and cognitive configurations [15,16]. From a public health perspective, these findings support a data-driven stratification of vaccine hesitancy patterns. The identified profiles suggest that individuals differ not only in the level of hesitancy but also in the underlying configuration of attitudes, safety concerns, and complacency. This supports the development of targeted interventions, where messaging and engagement strategies are adapted to specific behavioral profiles rather than applied uniformly across the population.

A contribution of this study is the incorporation of a continuum-based perspective in assessing vaccine hesitancy. Attitudes are tracked as they shift across vaccination phases, moving beyond the traditional approach in much of the literature, which often treats hesitancy as a static phenomenon [32,33]. This perspective is particularly important for public health, as it highlights the need for interventions that remain flexible and responsive even in the same population, capable of addressing emerging concerns or misinformation arising between primary vaccination and booster doses.

The observed heterogeneity in vaccine attitudes has several practical implications. Messaging campaigns that communicate the benefits and safety profiles of booster doses, reinforce collective responsibility, and directly counter prevalent misconceptions could be prioritized for groups with marked increases in hesitancy. Second, the identification of distinct attitudinal profiles supports the adoption of precision public health strategies, wherein communication, outreach, and support are tailored to align with the specific belief and behavior patterns of different subgroups [6]. Third, continuous monitoring of attitude changes may enable early identification of emerging hesitancy and timely intervention.

Our findings should be interpreted in light of several limitations. First, the reliance on self-reported survey data introduces potential bias, including social desirability which may lead to misestimation of both attitudes and behaviors [10]. Second, the sample is restricted to university students, which limits generalizability to other populations with differing age, educational, or socio-cultural profiles [2]. Third, while the predictive models incorporate a comprehensive set of behavioral and psychological variables, unmeasured factors such as social network influence, political affiliation, and exposure to misinformation may also contribute to booster hesitancy but were not captured in this study. Fourth, operationalizing susceptibility as the difference in hesitancy between primary and booster doses captures only two temporal points, restricting insight into longer-term trends and fluctuations in attitudes. Fifth, although clustering analysis identifies subgroups, these profiles may be context-specific and require validation in independent populations before broader application. Sixth, while machine learning interpretability tools provide insights into feature importance, they do not establish causal relationships; thus, the identified predictors should be interpreted as correlates rather than definitive determinants.

Finally, although the present study identifies distinct behavioral profiles and supports stratified interpretations of vaccine hesitancy change, it does not include direct measures of key mechanisms known to shape communication effectiveness in young adult populations. In particular, variables such as trust in information sources (e.g., healthcare providers or institutional authorities), exposure to specific media channels, media literacy, and peer or social normative influence were not available in the dataset. Consequently, the analysis cannot directly assess how these factors mediate or moderate responsiveness to communication strategies. This limits the ability to draw mechanistic conclusions regarding intervention effectiveness, and the identified profiles should therefore be interpreted as behavioral stratifications rather than communication-driven pathways.

Future research should examine changes in booster hesitancy longitudinally, explore the impact of targeted interventions on high-risk profiles, and consider integrating real-time indicators such as exposure to social media, media literacy, and peer or social normative influence. Extending similar analyzes to broader age groups, geographic settings, and culturally diverse populations would deepen understanding of the multilevel determinants of increased vaccine hesitancy.

## 6. Conclusions

This study shows that booster vaccine hesitancy among university students is primarily associated with modifiable attitudes and behaviors rather than demographic characteristics. XGBoost models accurately captured changes in hesitancy and revealed nuanced, individual-level patterns, while clustering analysis uncovered three distinct student profiles, ranging from minimal increases in hesitancy to consistently negative attitudes with pronounced safety concerns. These insights may inform targeted public health strategies: interventions should focus on reducing complacency, reinforcing confidence in vaccine safety, and tailoring communication to specific student profiles. Prioritizing the most susceptible subgroups may support the development of targeted public health strategies in comparable settings. More broadly, this work demonstrates the potential of machine learning to uncover complex, nonlinear relationships among predictors of vaccine attitudes, offering a scalable framework to monitor and address hesitancy in diverse populations and future vaccination campaigns.

## Figures and Tables

**Table 1:** Demographic characteristics of participants stratified by increased (+) and decreased (−) ΔVH.

Demographic Characteristics	ΔVH (−) (n = 1345)			ΔVH (+) (n = 3108)		
	n	%	95% CI	n	%	95% CI
**Gender**						
Women	797	59.3	56.6–61.9	1750	56.3	54.6–58.0
Man	471	35.0	32.5–37.6	1218	39.2	37.5–40.9
*Others*	49	3.6	2.8–4.8	77	2.5	2.0–3.1
Prefer not to say	28	2.1	1.4–3.0	63	2.0	1.6–2.6
**Age Groups**						
14–22	1071	79.6	77.4–81.7	2671	85.9	84.7–87.1
23–29	224	16.7	14.8–18.7	380	12.2	11.1–13.4
30–39	30	2.2	1.6–3.2	45	1.4	1.1–1.9
40 or more	20	1.5	1.0–2.3	12	0.4	0.2–0.7
**Degree**						
Undergraduate year 1	265	19.7	17.7–21.9	670	21.6	20.1–23.0
Undergraduate year 2	264	19.6	17.6–21.8	642	20.7	19.3–22.1
Undergraduate year 3	327	24.3	22.1–26.7	793	25.5	24.0–27.1
Undergraduate year 4+	401	29.8	27.4–32.3	857	27.6	26.0–29.2
Master’s student	49	3.6	2.8–4.8	100	3.2	2.7–3.9
PhD	27	2.0	1.4–2.9	37	1.2	0.9–1.6
*Others*	12	0.9	0.5–1.6	9	0.3	0.2–0.5
**Faculty**						
Arts	277	20.6	18.5–22.8	566	18.2	16.9–19.6
Engineering	306	22.8	20.6–25.1	771	24.8	23.3–26.4
Environment	102	7.6	6.3–9.1	248	8.0	7.1–9.0
Health	237	17.6	15.7–19.7	619	19.9	18.5–21.4
Mathematics	249	18.5	16.5–20.7	568	18.3	17.0–19.7
Science	174	12.9	11.2–14.8	336	10.8	9.8–12.0
**Status**						
Domestic	1165	86.6	84.7–88.3	2714	87.3	86.1–88.4
International	180	13.4	11.7–15.3	394	12.7	11.6–13.9
**Indigenous**						
No	1290	95.9	94.7–96.8	3032	97.6	97.0–98.0
Yes	24	1.8	1.2–2.6	39	1.3	0.9–1.7
Prefer not to say	31	2.3	1.6–3.3	37	1.2	0.9–1.6
**Immigration**						
Canadian citizen or Permanent resident	1156	85.9	84.0–87.7	2701	86.9	85.7–88.0
Temporary resident	152	11.3	9.7–13.1	355	11.4	10.4–12.6
Others	6	0.4	0.2–1.0	13	0.4	0.2–0.7
Prefer not to say	31	2.3	1.6–3.3	39	1.3	0.9–1.7
**Income**						
Less than $29,999	359	26.7	24.4–29.1	766	24.6	23.2–26.2
$30,000–59,999	110	8.2	6.8–9.8	303	9.7	8.8–10.8
$60,000–89,999	99	7.4	6.1–8.9	281	9.0	8.1–10.1
Greater than $90,000	339	25.2	23.0–27.6	753	24.2	22.8–25.8
Don’t know	211	15.7	13.8–17.7	548	17.6	16.3–19.0
Prefer not to say	227	16.9	15.0–19.0	457	14.7	13.5–16.0
**Marital Status**						
Single	1191	88.6	86.7–90.1	2866	92.2	91.2–93.1
Married	39	2.9	2.1–3.9	60	1.9	1.5–2.5
Others	70	5.2	4.1–6.5	108	3.5	2.9–4.2
Prefer not to say	45	3.3	2.5–4.4	74	2.4	1.9–3.0
**Employment**						
Employed	667	49.6	46.9–52.3	1554	50.0	48.2–51.8
Unemployed	623	46.3	43.7–49.0	1434	46.1	44.4–47.9
Prefer not to say	55	4.1	3.2–5.3	120	3.9	3.2–4.6

**Table 2:** 5-Fold Cross-Validation Performance of Regressors for Predicting Changes in Vaccine Hesitancy (ΔVH).

Model	$R^{2}$	MSE	MAE
GB	0.958 ± 0.003	10.19 ± 0.61	2.20 ± 0.07
XGBoost Regressor	0.977 ± 0.002	5.57 ± 0.52	1.51 ± 0.07

**Figure A1:**
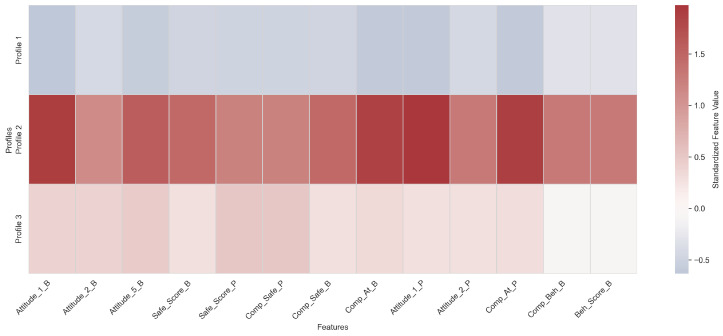
Standardized feature means for Gaussian Mixture Model-derived profiles among participants with ΔVH≤0.

## Data Availability

Data will be available upon request.

## Abbreviations

The following abbreviations are used in this manuscript:

COVID-19	Coronavirus Disease 2019
VH	Vaccine Hesitancy
ΔVH	Change in Vaccine Hesitancy between primary vaccination and booster dose
GB	Gradient Boosting
XGBoost	Extreme Gradient Boosting
SHAP	SHapley Additive exPlanations
GMM	Gaussian Mixture Model
PCA	Principal Component Analysis
MSE	Mean Squared Error
MAE	Mean Absolute Error
$R^{2}$	Coefficient of Determination
BIC	Bayesian Information Criterion
CVHS	COVID-19 Vaccine Hesitancy Scale
WHO	World Health Organization

## Appendix A

### Characterization of Participants with Non-Positive Changes in Vaccine Hesitancy

To provide a full-context comparison of vaccine hesitancy patterns, we conducted a supplementary analysis including participants with non-positive changes in hesitancy (ΔVH≤0). This group comprises individuals whose hesitancy remained stable or decreased between the primary vaccination series and the booster dose.

Figure A1 presents the standardized feature profiles for the resulting Gaussian Mixture Model solution. The analysis yields three profiles with a structure comparable to that reported in Section 3, preserving the same ordering across constructs but with lower overall levels of vaccine hesitancy-related measures.

These results indicate that the clustering structure identified in Section 3 reflects stable latent behavioral phenotypes that generalize beyond the ΔVH>0 subgroup, while the ΔVH≤0 population represents a downward-shifted manifestation of the same underlying structure.
